# Supplementary material for: Allergen Diversity and Abundance in Different Tissues of the Redclaw Crayfish (Cherax quadricarinatus)
Source: Foods. 2024 Jan 19;13(2):315. doi: 10.3390/foods13020315 (PMC10815050; doi:10.3390/foods13020315)
Supplement: Supplementary file 1 [file foods-13-00315-s001.zip › Supplementary Tables.pdf]

Supplementary Tables

Supplementary Table S1: Selected Patients from Adult and Paediatric Populations. Patients were either adults or paediatrics and were shellfish-sensitive (to shrimp-mix) as found on ALEX assay chip. Patients who were negative to shrimp-mix were chosen as negative controls.

Patient Number	JCU ID	Sex	Adult/Paediatric (A/P)	Shrimp Positive? (Y/N)
**Shellfish-Sensitive Patients (to Shrimp-Mix)**				
1	JCU 6	F	P	Y
2	JCU 27	F	P	Y
3	JCU 31	M	P	Y
4	JCU 34	M	P	Y
5	JCU 40	M	P	Y
6	JCU 47	F	P	Y
7	JCU 48	M	P	Y
8	JCU 58	M	P	Y
9	JCU 72	M	P	Y
10	JCU 77	F	P	Y
11	JCU 87	M	P	Y
12	JCU 88	M	P	Y
13	JCU 93	F	P	Y
14	JCU 8	F	A	Y
15	JCU 14	M	A	Y
16	JCU 16	F	A	Y
17	JCU 19	F	A	Y
18	JCU 43	M	A	Y
19	JCU 50	F	A	Y
20	JCU 54	F	A	Y
21	JCU 55	M	A	Y
22	JCU 59	F	A	Y
23	JCU 60	F	A	Y
24	JCU 86	M	A	Y
**Control Patients (Negative to Shrimp-Mix)**				
1	JCU 10	F	A	N
2	JCU 33	M	A	N
3	JCU 69	F	A	N

Supplementary Table S2: IgE-Binding in Raw Claw Analysed Using Liquid Chromatography-Mass Spectrometry. Top four most abundant proteins found for bands 1 - 4, including molecular weight (kDa), protein ID (NCBI), proteins found in each group, relative abundance (iBAQ%), coverage (%), protein MW (kDa).

Band	MW of Band	Hit	Protein ID (NCBI)	Protein Name	Proteins in Group	Relative Abundance (iBAQ%)	Coverage (%)	MW of Protein
1	75 kDa	1	AFP23115.1	**Hemocyanin**	1	61.66%	31.5%	78 kDa
		2	XP_053640584.1	Hemocyanin subunit-like	51	11.84%	28.6%	78 kDa
		3	XP_053640579.1	Hemocyanin subunit-like	1	6.11%	29.9%	78 kDa
		4	XP_053639178.1	Hemocyanin-like	8	4.80%	29.3%	76 kDa
2	74 kDa	1	XP_053627537.1	**Hemocyanin B chain-like**	27	38.44%	48.8%	78 kDa
		2	XP_053645744.1	Arginine kinase	107	14.81%	36.2%	50 kDa
		3	XP_053639179.1	Hemocyanin A chain-like	1	9.56%	17.6%	64 kDa
		4	AFP23115.1	Hemocyanin	1	6.96%	37.2%	78 kDa
3	37 kDa	1	XP_053645744.1	**Arginine kinase**	107	78.21%	42.2%	50 kDa
		2	XP_053649889.1	Fructose-biphosphate aldolase	7	7.80%	65.2%	40 kDa
		3	XP_053648131.1	Tropomyosin isoform X17	9	2.34%	60.9%	33 kDa
		4	sp|P14208.4|KARG_HOMGA	Arginine kinase	7	1.91%	30.9%	40 kDa
4	36 kDa	1	XP_053645744.1	**Arginine kinase**	107	70.25%	36.2%	50 kDa
		2	XP_045608276.1	Four and a half LIM domains protein 2-like isoform X1	13	5.89%	13.4%	39 kDa
		3	prf|671058A	Glyceraldehyde-3-phosphate dehydrogenase	1	3.29%	15.9%	36 kDa
		4	XP_053649889.1	Fructose-bisphosphate aldolase	7	2.59%	2.59%	40 kDa

Supplementary Table S3: IgE-Binding in Heated Claw Analysed Using Liquid Chromatography-Mass Spectrometry. Top four most abundant proteins found for bands 1 - 4, including molecular weight (kDa), protein ID (NCBI), proteins found in each group, relative abundance (iBAQ%), coverage (%), protein mass (kDa).

Band	MW of Band	Hit	Protein ID (NCBI)	Protein Name	Proteins in Group	Relative Abundance (iBAQ%)	Coverage (%)	MW of Protein
1	75 kDa	1	AFP23115.1	**Hemocyanin**	1	29.25%	38.4%	78 kDa
		2	XP_053627537.1	Hemocyanin B chain-like	27	15.12%	37.6%	78 kDa
		3	XP_053648131.1	Tropomyosin isoform X17	9	12.00%	35.2%	32 kDa
		4	XP_053640584.1	Hemocyanin subunit-like	51	8.89%	23.3%	78 kDa
2	37 kDa	1	XP_053648131.1	**Tropomyosin isoform X17**	9	94.6%	81%	33 kDa
		2	XP_053648135.1	Tropomyosin isoform X21	1	2.37%	83.1%	33 kDa
		3	XP_053641032.1	Troponin T isoform X16	2	0.94%	17.7%	33 kDa
		4	XP_042241082.1	Tropomyosin isoform X24	1	0.83%	80.6%	33 kDa
3	20 kDa	1	XP_053629360.1	**Myosin light chain 2**	6	50.82%	13.8%	19 kDa
		2	XP_053648994.1	Myosin light chain 2	3	38.08%	12.3%	19 kDa
		3	XP_053634537.1	Sarcoplasmic calcium-binding protein isoform X3	1	3.42%	6.7%	22 kDa
		4	XP_053648131.1	Tropomyosin isoform X17	9	1.97%	81%	33 kDa
4	17 kDa	1	XP_053649409.1	**Troponin C isoform X2**	8	56.36%	59.4%	17 kDa
		2	XP_053642032.1	Uncharacterised protein LOC128695440	1	11.22%	24.8%	16 kDa
		3	XP_053641032.1	Troponin T isoform X16	2	5.27%	9.9%	33 kDa

Supplementary Table S4: IgE-Binding in Raw Tail Analysed Using Liquid Chromatography-Mass Spectrometry. Top four most abundant proteins found for bands 1 - 3, including molecular weight (kDa), protein ID (NCBI), proteins found in each group, relative abundance (iBAQ%), coverage (%), protein mass (kDa).

Band	MW of Band	Hit	Protein ID (NCBI)	Protein Name	Proteins in Group	Relative Abundance (iBAQ%)	Coverage (%)	MW of Protein
1	75 kDa	1	AFP23115.1	**Hemocyanin**	1	20.42%	42.6%	78 kDa
		2	XP_053645744.1	Arginine kinase	107	10.69%	33.9%	50 kDa
		3	XP_053631598.1	Muscle-specific protein 300 kDa-like	4	8.38%	59.1%	70 kDa
		4	XP_053627537.1	Hemocyanin B chain-like	27	6.32%	41%	78 kDa
2	37 kDa	1	XP_053645744.1	**Arginine kinase**	107	70.64%	53.7%	50 kDa
		2	XP_053648131.1	Tropomyosin isoform X17	9	13.53%	64.4%	33 kDa
		3	XP_053649889.1	Fructose-bisphosphate aldolase-like	7	4.90%	65.8%	40 kDa
		4	sp|P14208.4|KARG_HOMGA	Arginine kinase	7	2.41%	37.4%	40 kDa
3	37 kDa	1	XP_053645744.1	**Arginine kinase**	107	72.89%	49.7%	50 kDa
		2	XP_053648131.1	Tropomyosin isoform X17	9	11.64%	65.1%	33 kDa
		3	XP_053649889.1	Fructose-bisphosphate aldolase-like	7	2.22%	48.2%	40 kDa
		4	prf||671058A	Glyceraldehyde-3-phosphate dehydrogenase	1	2.22%	32.1%	36 kDa

Supplementary Table S5: IgE-Binding in Heated Tail Analysed Using Liquid Chromatography-Mass Spectrometry**.** Top four most abundant proteins found for bands 1 – 12, including molecular weight (kDa), protein ID (NCBI), proteins found in each group, relative abundance (iBAQ%), coverage (%), protein mass (kDa).

Band	MW of Band	Hit	Protein ID (NCBI)	Protein Name	Proteins in Group	Relative Abundance (iBAQ%)	Coverage (%)	MW of Protein
1	100 kDa	1	XP_053645272.1	**Myosin heavy chain**	21	28.58%	34.8%	218 kDa
		2	XP_053645271.1	Myosin heavy chain	5	17.56%	33.5%	218 kDa
		3	XP_053648131.1	Tropomyosin isoform X17	9	13.57%	53.2%	33 kDa
		4	XP_053646515.1	Proteoglycan 4-like	1	5.73%	23.7%	67 kDa
2	75 kDa	1	XP_053645272.1	**Myosin heavy chain**	21	27.34%	34.2%	218 kDa
		2	XP_053648131.1	Tropomyosin isoform X17	9	26.83%	60.2%	33 kDa
		3	XP_053645271.1	Myosin heavy chain	5	10.74%	31.8%	2198kDa
		4	AFP23115.1	Hemocyanin	1	5.83%	26.9%	78 kDa
3	57 kDa	1	XP_053648131.1	**Tropomyosin isoform X17**	9	41.06%	54.9%	33 kDa
		2	XP_053645272.1	Myosin heavy chain	21	26.75%	28.4%	218 kDa
		3	XP_053645271.1	Myosin heavy chain	5	13.99%	30.0%	218 kDa
		4	XP_053632103.1	PDZ and LIM domain protein Zasp-like isoform X15	40	3.23%	19.1%	127 kDa
4	50 kDa	1	XP_053648131.1	**Tropomyosin isoform X17**	9	95.28%	81.7%	33 kDa
		2	XP_053629360.1	Myosin light chain 2	6	0.87%	23.7%	19 kDa
		3	XP_053641032.1	Troponin T	2	0.75%	27.7%	33 kDa
		4	XP_053634537.1	Sarcoplasmic calcium-binding protein	1	0.34%	45.6%	22 kDa
5	38 kDa	1	XP_053648131.1	**Tropomyosin isoform X17**	9	93.17%	83.5%	33 kDa
		2	XP_053641032.1	Troponin T isoform X16	2	0.82%	31.6%	33 kDa

Band	MW of Band	Hit	Protein ID (NCBI)	Protein Name	Proteins in Group	Relative Abundance (iBAQ%)	Coverage (%)	MW of Protein
		3	XP_053634537.1	Sarcoplasmic calcium-binding protein isoform X3	1	0.77%	46.1%	22 kDa
		4	XP_053645744.1	Arginine kinase isoform X3	107	0.46%	29.7%	50 kDa
6	37 kDa	1	XP_053648131.1	**Tropomyosin isoform X17**	9	98.14%	84.5%	33 kDa
		2	XP_053629360.1	Myosin light chain 2	6	0.75%	44.8%	19 kDa
		3	XP_053641032.1	Troponin T isoform X16	2	0.32%	35.8%	33 kDa
		4	XP_045130235.1	Tropomyosin isoform X23	3	0.27%	80.6%	33 kDa
7	31 kDa	1	XP_053648131.1	**Tropomyosin isoform X17**	9	71.36%	69.4%	33 kDa
		2	XP_053641032.1	Troponin T isoform X16	2	12.23%	31.6%	33 kDa
		3	XP_053646012.1	Troponin isoform X7	80	1.57%	42.4%	28 kDa
		4	XP_053650292.1	Protein lethal(2) essential for life-like	1	1.44%	52%	27 kDa
8	25 kDa	1	XP_053648131.1	**Tropomyosin isoform X17**	9	28.13%	64.4%	33 kDa
		2	XP_053652529.1	Adenylate kinase isoenzyme-1-like isoform X2	16	23.63%	71.3%	22 kDa
		3	XP_053629360.1	Myosin light chain 2	6	15.91%	44.8%	19 kDa
		4	XP_053641032.1	Troponin T isoform X16	2	4.43%	26.6%	33
9	20 kDa	1	XP_053629360.1	**Myosin light chain 2**	6	78.87%	89.1%	19 kDa
		2	XP_053634537.1	Sarcoplasmic calcium-binding protein isoform X3	1	10.05%	70.5%	22 kDa
		3	XP_053634536.1	Sarcoplasmic calcium-binding	1	4.91%	73.6%	22 kDa

Band	MW of Band	Hit	Protein ID (NCBI)	Protein Name	Proteins in Group	Relative Abundance (iBAQ%)	Coverage (%)	MW of Protein
				protein isoform X2				
		4	XP_053641032.1	Troponin T isoform X16	2	1.57%	25.2%	33 kDa
10	18 kDa	1	XP_053629360.1	**Myosin light chain 2**	6	50.33%	62.1%	19 kDa
		2	XP_053634537.1	Sarcoplasmic calcium-binding protein isoform X3	1	12.60%	62.7%	22 kDa
		3	XP_053641032.1	Troponin T isoform X16	2	8.84%	22.3%	33 kDa
		4	XP_053648131.1	Tropomyosin isoform X17	9	5.52%	59.2%	33 kDa
11	17 kDa	1	XP_053656247.1	**Myosin light chain 1 isoform X3**	18	45.10%	57.9%	15 kDa
		2	XP_053635594.1	Sodium/calcium exchanger regulatory protein 1	8	7.97%	57.4%	16 kDa
		3	XP_053629360.1	Myosin light chain 2	6	6.15%	35.6%	19 kDa
		4	XP_053632402.1	2-iminobutanoate/2-iminopropanoate deaminase	9	5.37%	39.7%	14 kDa
12	16 kDa	1	XP_053656247.1	**Myosin light chain 1 isoform X3**	18	27.86%	57.9%	15 kDa
		2	XP_053649414.1	Troponin C isoform X2	2	14.48%	77.3%	17 kDa
		3	XP_053629360.1	Myosin light chain 2	6	13.87%	35.6%	19 kDa
		4	sp|P06707.1|TNNC1_ASTLP	Troponin C isoform X2	6	6.53%	31.3%	17 kDa

Supplementary Table S6: IgE-Binding in Raw Cephalothorax Analysed Using Liquid Chromatography-Mass Spectrometry. Top four most abundant proteins found for bands 1 - 5, including molecular weight (kDa), protein ID (NCBI), proteins found in each group, relative abundance (iBAQ%), coverage (%), protein mass (kDa).

Extract	MW of Band	Hit	Protein ID (NCBI)	Protein Name	Proteins in Group	Relative Abundance (iBAQ%)	Coverage (%)	MW of Protein
1	75 kDa	1	AFP23115.1	**Hemocyanin**	1	29.67%	38.7%	78 kDa
		2	XP_053627537.1	Hemocyanin B chain	27	16.51%	39%	78 kDa
		3	AAG17936.1	Vitellogenin	15	12.31%	31.2%	292 kDa
		4	XP_053640584.1	Hemocyanin subunit	51	11.17%	33.3%	78 kDa
2	57 kDa	1	XP_053627537.1	**Hemocyanin B chain**	27	19.59%	39.3%	78 kDa
		2	AFP23115.1	Hemocyanin	1	16.58%	34.1%	78 kDa
		3	AAG17936.1	Vitellogenin	15	13.08%	32.8%	292 kDa
		4	XP_053640579.1	Hemocyanin subunit	1	10.67%	35.9%	78 kDa
3	37 kDa	1	XP_053648131.1	**Tropomyosin isoform X17**	9	46.47%	62.3%	33 kDa
		2	XP_053640001.1	Mannan endo-1,4-beta-mannosidase	1	18.52%	22.3%	43 kDa
		3	XP_053642615.1	Vitellogenin	2	3.53%	21.1%	310 kDa
		4	XP_053656677.1	Mannan endo-1,4-beta-mannosidase	1	2.60%	19.4%	41 kDa
4	19 kDa	1	XP_053634537.1	**Sarcoplasmic calcium-binding protein isoform X3**	1	46.73%	62.7%	22 kDa
		2	XP_053634538.1	Sarcoplasmic calcium-binding protein isoform X4	1	8.94%	47.2%	22 kDa
		3	XP_053648131.1	Tropomyosin isoform X17	9	8.75%	55.6%	33 kDa
		4	XP_053634536.1	Sarcoplasmic calcium-binding	1	5.03%	57.5	22 kDa

Extract	MW of Band	Hit	Protein ID (NCBI)	Protein Name	Proteins in Group	Relative Abundance (iBAQ%)	Coverage (%)	MW of Protein
				protein isoform X2				
5	17 kDa	1	XP_053635594.1	**Sodium/calcium exchanger regulatory protein 1**	8	9.46%	57.4%	16 kDa
		2	XP_053648131.1	Tropomyosin isoform X17	9	7.59%	54.9%	33 kDa
		3	XP_053651723.1	Fatty acid-binding protein	1	5.98%	36.9%	14 kDa
		4	XP_053639178.1	Hemocyanin	8	5.49%	11.8%	76 kDa

Supplementary Table S7: IgE-Binding in Heated Cephalothorax Analysed Using Liquid Chromatography-Mass Spectrometry. Top four most abundant proteins found for bands 1 and 2, including molecular weight (kDa), protein ID (NCBI), proteins found in each group, relative abundance (iBAQ%), coverage (%), protein mass (kDa).

Extract	MW of Band	Hit	Protein ID (NCBI)	Protein Name	Proteins in Group	Relative Abundance (iBAQ%)	Coverage (%)	MW of Protein
1	75 kDa	1	AFP23115.1	**Hemocyanin**	1	29.28%	34.1%	78 kDa
		2	AAG17936.1	Vitellogenin	15	16.58%	29.2%	292 kDa
		3	XP_053627537.1	Hemocyanin B chain	27	10.97%	31.6%	78 kDa
		4	XP_053648131.1	Tropomyosin isoform X17	9	8.67%	44.4%	33 kDa
2	37 kDa	1	XP_053648131.1	**Tropomyosin isoform X17**	9	94.25%	70.1%	33 kDa
		2	XP_053636460.1	Oplophorus-luciferin 2-monooxygenaseisoform X1	2	2.52%	11.3%	38 kDa
		3	XP_042241082.1	Tropomyosin isoform X24	1	0.37%	70.1%	33 kDa
		4	KAG0711415.1	Succinate-CoA ligase	9	0.24%	10.1%	13 kDa
